# Effectiveness of tailored digital health interventions for mental health at the workplace: A systematic review of randomised controlled trials

**DOI:** 10.1371/journal.pdig.0000123

**Published:** 2022-10-21

**Authors:** Thirimon Moe-Byrne, Jessie Shepherd, Dorota Merecz-Kot, Marjo Sinokki, Päivi Naumanen, Leona Hakkaart-van Roijen, Christina Van Der Feltz-Cornelis

**Affiliations:** 1 Department of Health Sciences, University of York, York, United Kingdom; 2 Institute of Psychology, Lodz, Lodz, Poland; 3 Turku Centre for Occupational Health, University of Turku, Turku, Finland; 4 Erasmus School of Health Policy and Management (ESHPM), Erasmus University Rotterdam, Rotterdam, the Netherlands; 5 Hull York Medical School, University of York, York, United Kingdom; 6 Institute of Health Informatics, University College London, London, United Kingdom; University of Bayreuth: Universitat Bayreuth, GERMANY

## Abstract

Mental health problems in the workplace are common and have a considerable impact on employee wellbeing and productivity. Mental ill-health costs employers between £33 billion and £42 billion a year. According to a 2020 HSE report, roughly 2,440 per 100,000 workers in the UK were affected by work-related stress, depression, or anxiety, resulting in an estimated 17.9 million working days lost. We performed a systematic review of randomised controlled trials (RCTs) to assess the effect of tailored digital health interventions provided in the workplace aiming to improve mental health, presenteeism and absenteeism of employees. We searched several databases for RCTs published from 2000 onwards. Data were extracted into a standardised data extraction form. The quality of the included studies was assessed using the Cochrane Risk of Bias tool. Due to the heterogeneity of outcome measures, narrative synthesis was used to summarise the findings. Seven RCTs (eight publications) were included that evaluated tailored digital interventions versus waiting list control or usual care to improve physical and mental health outcomes and work productivity. The results are promising to the advantage of tailored digital interventions regarding presenteeism, sleep, stress levels, and physical symptoms related to somatisation; but less for addressing depression, anxiety, and absenteeism. Even though tailored digital interventions did not reduce anxiety and depression in the general working population, they significantly reduced depression and anxiety in employees with higher levels of psychological distress. Tailored digital interventions seem more effective in employees with higher levels of distress, presenteeism or absenteeism than in the general working population. There was high heterogeneity in outcome measures, especially for work productivity; this should be a focus of attention in future studies.

## Author summary

Work-related stress has become a major issue at the work place. It is associated with increased physical and mental health risk. Work stress is also associated with long term economic impact due to absenteeism and presenteeism. There have been several workplace interventions to tackle this issues, with varying results. In recent years, digital health interventions have become increasingly popular to promote employees’ physical and mental wellbeing. Some have shown moderate effects improving employees’ psychological wellbeing. However, most interventions were not tailored to the individual employee. So far, no review has explored the effect of tailored digital interventions addressing stress in the workplace compared to usual care. Also, no reviews have taken blended e-health models into account explicitly. This systematic review seeks to address this gap. We found that tailored digital interventions did not reduce anxiety or depression in the general working population but they significantly reduced anxiety in employees with higher levels of psychological distress. However, as the outcomes measured were so diverse, it is difficult to draw conclusions. There is a need for uniformity in the use of assessment tools and outcomes reporting in future research, especially in terms of work productivity.

## Introduction

### Background

Mental ill-health conditions such as depression, anxiety, and stress are common in the workplace. They impact employee wellbeing, productivity, absenteeism (sickness absence), and presenteeism [1,2]. Presenteeism is working with difficulty to do the tasks at hand [3]. Originally coined as “showing up at work while being sick” [4] because of chronic medical conditions [5] or because of work or personal characteristics, the emphasis in interpretation has shifted toward worker slowdowns in general and the economic costs associated with that [6]. The prevalence of presenteeism is high, amounting to an average of 40% in a survey conducted amongst workers in 34 countries [7–9].

Globally, mental health problems as a cause of the burden of illness are on the rise and account for a fifth of all years of living disabled [10]. The Global Burden of Disease study reported that in 2017, an estimated 792 million people are affected by mental ill-health worldwide [11] and a 2018 Lancet Commission report on mental health estimates mental health disorders will cost the global economy 16 trillion US dollars by 2030 [12].

Mental ill-health costs employers between £33 billion and £42 billion a year, with an annual cost to the UK economy of between £74 billion and £99 billion [13]. According to a Health and Safety Executive report in 2020 there were an estimated 828,000 workers affected by work-related stress, depression, or anxiety; these three conditions are responsible for 51% of all cases of work-related ill health and 55% of all days lost due to work-related ill health in the UK [14,15]. Work-related stress is a significant risk factor for a range of physical and mental health conditions such as cardiovascular disease, depression and related mortality [16,17]. In addition, a 2020 survey undertaken on 3,614 UK workers reported that 41% of employees surveyed have experienced mental health symptoms that were caused or worsened by work and 51% said their symptoms were due to pressure they experienced at work [18].

Understanding how employee health impacts productivity has advanced substantially in the past two decades [19–22]. According to a survey conducted in 2019, 69% of UK line managers were aware that supporting employee wellbeing is a core management requirement; however, only 13% have received any form of wellbeing or mental health training [23].

Non-digital workplace interventions have been developed to promote employees’ wellbeing and potentially prevent or treat mental disorders [24] and a few studies showed some positive results with workplace interventions such as cognitive-behavioural therapy, mindfulness and relaxation [24–28]. Also, one review which explored the effect on absenteeism and productivity found a small number of studies that suggested some effect for short interventions [29].

In the past decade, the advancement of digital technology has led to the creation of digital health interventions in physical and mental health. Nowadays, web based and smartphone based digital health interventions have become increasingly popular within the health industry due to their easy implementation [30]. In addition, digital interventions have the potential to be scaled up to provide care to large populations and could offer greater anonymity and stigma reduction compared to traditional face-to-face interventions [16,31]. Several reviews published in 2017 and 2018 found that digital interventions could effectively improve employees’ psychological wellbeing and reduce stress at the workplace. They explored the impact of digital interventions without further support from health professionals on health-related outcomes, and most of the interventions examined were not tailored to the individual employee [30,32,33]. Tailoring in this context differs from mode tailoring as described elsewhere [34]. It indicates an automated process that adapts the intervention based upon the employee’s input, for example, by applying an algorithm to the individual responses from the baseline questionnaire to generate specific feedback and provide modules relevant to the user. Also, blended eHealth interventions delivered by healthcare professionals combined with a digital intervention tailoring to the treatment is now available. So far, their application and effect in the workplace setting has not been explored.

This systematic review seeks to evaluate the impact of tailored digital health interventions aiming to improve mental health, stress-related physical symptoms, presenteeism and absenteeism in the workplace in employees in case of mental health issues or work-stress.

## Methods

### Protocol and registration

The review protocol has been registered in the PROSPERO database under ID CRD42021213292 [35]. This review complies with the Preferred Reporting Items for Systematic Reviews and Meta-Analysis (PRISMA) guidelines (See S3 Appendix) [36]. This study was funded by the European Union’s Horizon 2020 research and innovation program under grant agreement number 848180.

### Inclusion criteria

RCTs, including pilot and feasibility trials, with individual or cluster randomisation and stepped wedge trial designs (SWTD), were eligible if they assessed the impact of digital health interventions compared with standard guidance or waiting list control. The participants had to be employees aged 16 years and above, and the interventions had to be provided in or via the workplace setting.

Included studies had to report interventions that were delivered using digital technology. Digital technology includes digital decision aids or materials delivered through a computer, tablet, smartphone or email. This material could be delivered as a website, app or downloadable software. This digital intervention could be combined with further support such as group sessions, individual counselling, or direct feedback from a health professional (such as an occupational physician, a psychotherapist, a coach in the workplace, or psychiatric consultation), so-called blended e-Health interventions. This further support was not necessary for inclusion, however. The studies had to report on mental health outcomes or work-stress. Additionally, they could report on physical outcomes, presenteeism or absenteeism. We have also included studies related to insomnia as sleep-wake disorders are a classification in the Diagnostic Statistical Manual of Mental disorders (DSM-5) [37]. To be included, a digital intervention needed to have some tailored feedback and content for the individual employee. Feedback from an outside therapist or group, not included in the digital intervention, did not meet this criterion. Also, online training or monitoring without tailoring and personalisation would not meet this criterion.

### Exclusion criteria

Studies were excluded if they reported interventions that were not tailored or personalised in some way to the individual employee; if the feedback provided to the participants was not tailored or individualised in some way; or if the participants were provided with material to practice on their own, such as mindful breathing or meditation, with no tailoring based upon client assessment and symptomatology. Feedback from an outside therapist or group, not included in the digital intervention itself, did not count as relevant here.

The studies were also excluded if the interventions solely addressed addiction or consisted of online training/teaching or online education without tailoring. Interventions aimed at physical outcomes, productivity, performance, presenteeism, absenteeism or lifestyle interventions without mental health or work-stress outcomes were also excluded. Studies reporting on cost-effectiveness were out of scope.

### Search strategy

Relevant studies were identified by searching the following electronic databases: MEDLINE, EMBASE, PsycINFO, and The Cochrane Central Register of Controlled Trials (CENTRAL) published from 2000 onwards. We searched Google Scholar for grey literatures and the reference lists of all included articles and relevant systematic reviews were also checked to identify potentially eligible studies. Experts who published in the domain of workplace stress, mental health and digital interventions were contacted to suggest any articles or grey literature that they deemed relevant. We did not apply any language restrictions (See S1 Appendix for a complete list of search terms and combinations). The search was supported by the Centre for reviews and Dissemination (CRD) at the University of York.

### Study selection

The studies retrieved from the searches were exported into EndNote. After deduplication, the rest of the studies were exported into Rayyan (Rayyan QCRI software for screening) [38]. The first 10% of the titles and abstracts of the studies were screened in duplicate by two independent researchers (TMB and JS) against the inclusion/exclusion criteria. We coded either 1 for inclusion or 0 for exclusion. We used Cohen’s Kappa statistic to calculate the percentage of positive agreement and negative agreement between the reserachers.

We considered the second round of 10% double screening if the kappa score was less than 0.8. However, we have achieved an excellent inter-rater agreement in our first round (Cohen’s Kappa score 0.96), and no potentially eligible studies were missed. Hence, two reviewers continued screening half of each of the remaining studies. Full texts of potentially eligible studies were then screened independently and in duplicate by two reviewers (TMB and JS). Disagreements were resolved through discussion to achieve consensus, and if necessary, a third reviewer (CFC) was consulted. In all cases, a consensus was reached.

### Data extraction

Data extraction was carried out using a standardised data extraction form. This extraction form was piloted (by filling in the first two studies) and refined. Data were extracted as follows: study design (e.g., RCT, Cluster randomisation or Stepped wedge), study participants (age, sex) and interventions (population, sample size, intervention/comparator details), outcome measures (outcome description and measures), results, effect size and findings. We also extracted the types of personalisation and tailoring used for participants and the theoretical background upon which the digital intervention materials were based, and created a table showing triage, personalisation, and intervention tailoring used by the individual trials, in the results section. Tailoring methods were extracted from each article, using the original authors language and terminology. Commonalities and overlaps were examined, this process was done inductively based upon the language used in the articles, and five categories of tailoring emerged. They are: 1) Algorithm used to triage based upon assessment or questionnaire scores, 2) Algorithm used for tailored Summarised Feedback, 3) Participant choice, 4) Use of automated messages to users, 5) Interventions blended with in-person support. Table 1 shows each of the five categories.

**Table 1:** Tailoring and Personalisation table.

Authors	Programmed Algorithm Used for Triage	Algorithm used for tailored Feedback	Participant Choice	Automated Messages	Blended with In-person Support
Billings et al. 2008 [43]	Use of assessment instrument to recommend content		Participants pick content they feel best fits their needs		
Bolier, et al., 2014 & Ketelaar et al., 2013 [48,49]	Material tailored based on user screening results	Provide personalised feedback			
Bostock et al. 2016 [47]	Material tailored based on user characteristics, goals, and sleep data			Automatic prompts and messages	
Ebert et al., 2016 [44]	Material tailored based on user responses while completing exercises		Participants can pick content they feel best fits their needs	Participant can chose to turn on automated messages	
Grime, 2004 [45]	Material and exercises are assigned at the end of each module and debriefed at the beginning of each	Provide tailored feedback and summaries			77% of participants received "Conventional Care" (p. 357) or counselling and medication
Volker et al., 2015 [50]	Use of assessment instrument to recommend modules				Support for participants from an occupational physician
Weber et al., 2019 [46]		Provide tailored feedback and summaries based on screening results and sleep data	Participants pick content they feel best fits their needs		

Data were extracted in duplicate by two reviewers independently (TMB and JS). Disagreements were resolved through consensus, and if necessary, a third reviewer (CFC) was consulted. Data from studies with multiple publications were extracted and reported as a single study. The study authors were contacted for further information when appropriate.

### Quality assessment

The quality of the RCTs was assessed according to the Cochrane Risk of Bias tool (low, high, uncertain) [39] supported by a standardised data extraction form. The tool assessed several sources of bias, including selection, performance, detection, attrition, and reporting bias. The clustered RCTs were assessed using the same tool but amended by adding the following sources of bias (recruitment bias, baseline imbalance, loss of clusters, incorrect analysis, and comparability with individual randomised trials) which are particular to the Clustered RCTs [40].

We produced the overall risk of bias assessment for each study (low, high, uncertain) based on reviewing all sources of bias identified in the individual trial. Two reviewers (TMB and JS) independently assessed the quality of the studies. Disagreements were resolved through consensus, and if necessary, a third reviewer (CFC) was consulted. The funnel plot to assess the publication bias was not conducted as there were less than ten trials in the review [41].

### Data analysis

Due to the lack of homogeneity across outcome measures, we did not consider formal meta-analysis a feasible option. Therefore, a narrative approach was used to summarise the findings as presented in tables in the Results section. We have also presented the percentage range for adherence and attrition in each group. We considered less than 25% as low, less than 50% as moderate and 50% and above as good.

## Results

### Study selection

**Fig 1:**
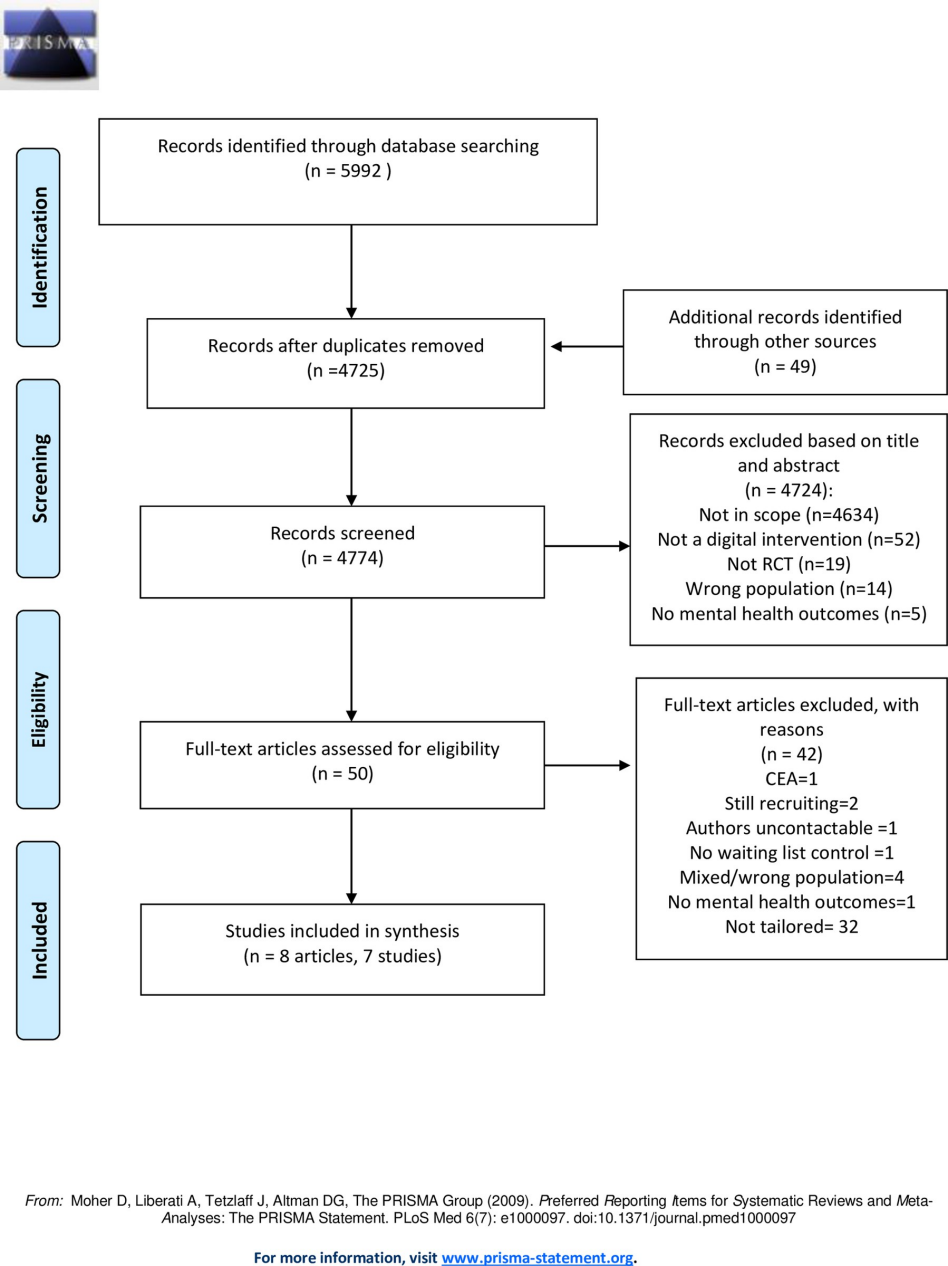
PRISMA 2009 Flow Diagram.

The study selection is shown in Fig 1 below. A total of 5992 publications were identified through the initial database search, and an additional 49 studies were identified through google scholar and citation search. After the removal of duplicates, 4774 publication titles and abstracts were screened. Fifty studies were eligible for a full-text review. We could not retrieve a full-text review of 1 study as the authors did not reply to our email [42]. Out of 50 studies, 42 were excluded because the interventions were not tailored to the individuals, reported no mental health outcomes, addressed a mixed or wrong population, had no waiting list control, the full text was not available, and some were ongoing trials or a cost-effectiveness study which was out of scope.

### Study characteristics

Eight publications (7 studies) met the inclusion criteria: five RCTs [43–46], one crossover RCT [47] and two cluster RCTs [48–50]. Two studies [48,49] were part of a larger trial [51] which used the same data to report different outcomes. One study was conducted in the USA [43], two studies (three publications) in the Netherlands [48–50], two in Germany [44,46] and two in the UK [45,47]. The total number of participants included in this review was 2008, with study sample sizes ranging from 48 to 532. The studies were conducted between 2004 and 2019.

### Participant characteristics

Four studies only included participants with particular characteristics. One study included only participants that screened positive for depression, somatisation, or anxiety based on the following assessment tools: the Patient Health Questionnaire for depression (PHQ-9), for somatisation (PHQ-15), and for Generalised Anxiety Disorder (GAD-7); this study also required that participants were on sick-leave between 4 and 26 weeks [50]. One study required participants to screen positive on the General Health Questionnaire (GHQ 12) [45] and to be sick-listed at least 4 weeks; and one required participants screening positive on the Perceived Stress Scale (PSS-10) [44]. A fourth study only included participants who self-identified as having poor sleep [47]. One study excluded participants if they were on sick leave for more than two weeks [48,49]. The remaining two studies did not report any specific inclusion/exclusion criteria [43,46].

Participants were recruited from a variety of workplaces. Four studies recruited the employees from organisations such as an insurance company, a technology company, or large corporations [43,44,47,50]. Two studies recruited healthcare professionals from local hospitals [45,48,49]. One study recruited employees from the private and public sector companies in three countries [46].

### Intervention characteristics

Three studies (four publications as mentioned above) included in this review designed their digital interventions to increase wellbeing and act as preventative interventions for mental health problems such as anxiety and depression, rather than treating these conditions [43,46,48,49]. One focused on work performance and provided guidance to the Occupational Physician to address any mental wellbeing issues together with the GP, as a blended eHealth model [50]. The length of the administration of the interventions ranged from four weeks to three months. All studies included in this review compared a web-based intervention, [43,44,48–50] a smartphone app intervention [46] and a combined web based and smartphone app intervention [47] with waiting list or usual guidance control. All the studies stated the use of Cognitive Behavioural Therapy (CBT) techniques as theoretical background for the materials chosen and content uploaded into the digital intervention. Additionally, two stated the use of Mindfulness-based content and materials [46,48,49]. Other studies used the transactional stress model or the job demands-resources (JDR) model as background [44,46].

Triage used in the application of digital support systems refers to the assessment by questionnaires to gauge the mental and or physical state of the participant, which informs the tailoring algorithm.

Table 1 below shows each of the five categories and which articles used which tailoring tool.

The use of an Algorithm refers to programmed instructions in the digital intervention that guide the assignment of materials or the generation of feedback. Digital interventions that use algorithms, automate, and personalise the feedback and content that a user sees. The algorithm guides content assignment based on the users’ feedback, assessment scores, actions, or behaviour within the digital intervention. Guidance can differ depending on the focus of the digital intervention. For example, with the Kelaa Mental Resilience App [46], the digital intervention used sensors built in users smartphones to track the users’ sleep and then provide detailed feedback. One study provided support from participants by occupational physicians who received instructions and decision support from the digital intervention, based upon the tailoring [50]. This was the only study evaluating a blended digital intervention. Two studies allowed digital intervention to be combined with regular counselling and medication, or “treatment as usual” [44,45]; this is not considered blended care as the real life treatment is not influenced by the digital intervention.

The characteristics for tailoring are shown in Table 1 and S2 Appendix.

### Outcome measures

Most studies reported a mix of psychological (such as depression, anxiety, and stress) and work-related outcome measures (such as absenteeism and presenteeism). Several studies reported physical measures: two studies reported outcomes relevant to sleeping problems [44,46] and one measured the amount of sleep and workplace performance [47]. One study reported physical symptoms related to somatisation [50] and the other two reported levels of physical health impairment using self-reported SF12 and SF 36 questionnaires [44,46]. However, none of the studies reported on long-term medical conditions such as diabetes or cardiovascular disease. Regarding the crossover trial, we only used first period data prior to crossover [47].

### Risk of bias

**Fig 2:**
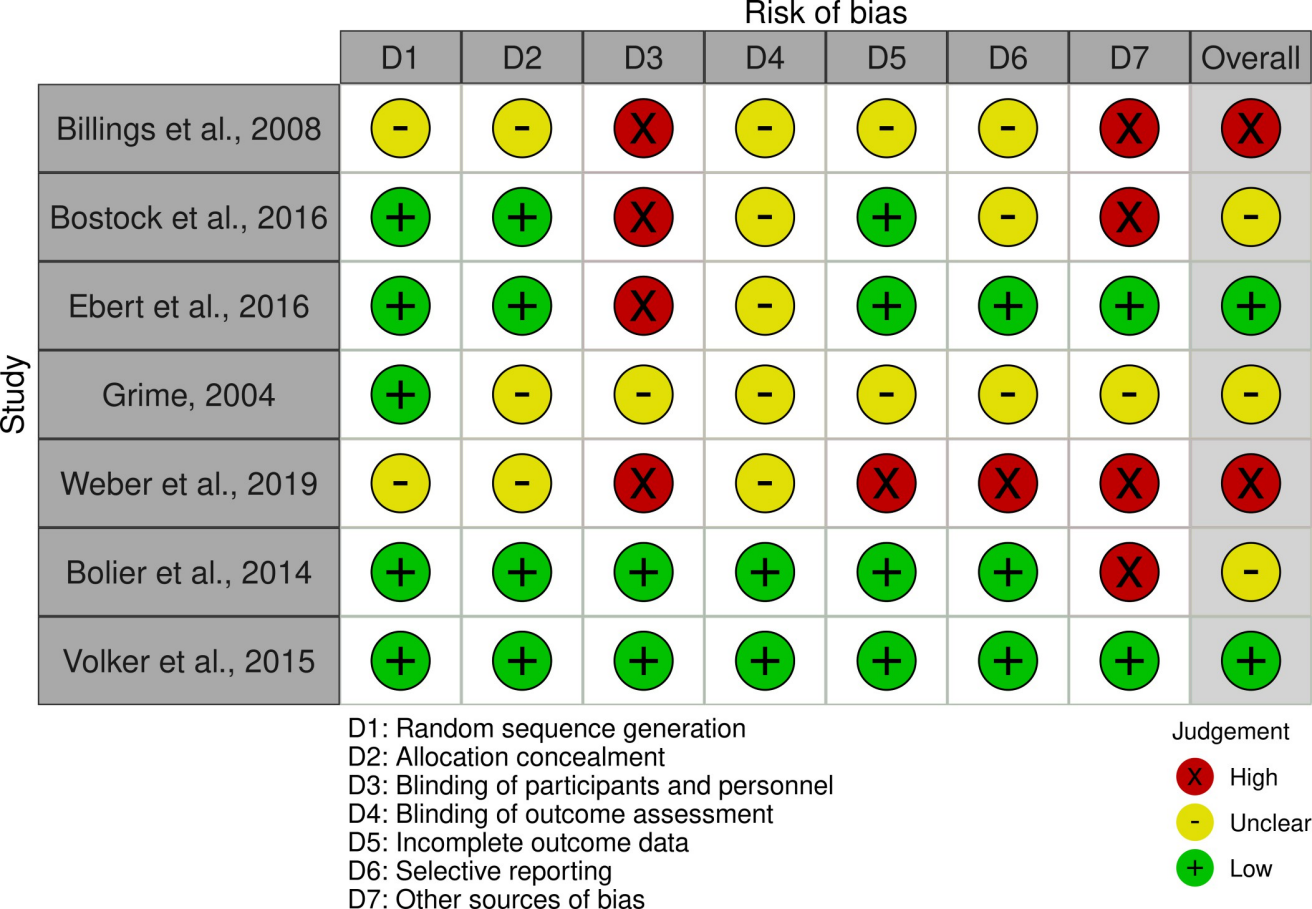
Risk of Bias Visualization Plot.

Table 2 and Fig 2 show full details of the risk of bias across all studies. Overall, two studies out of seven were judged to be at low risk of bias [44,50]. For the remaining four studies, three were judged to be at unclear risk of bias [45,47–49] and two were at high risk of bias [43,46].

**Table 2:** Risk of bias in included studies.

Author, Year	Trial design	Random sequence generation	Allocation concealment	Blinding of participants and personnel	Blinding of outcome assessment	Incomplete outcome data	Selective reporting	*Other sources of bias	Overall assessments
Billings et al., 2008 [43]	RCT	UC, randomisation method not reported	UC, randomisation method not reported	H, unable to blind due to the nature of the study	UC, not reported	UC, attrition rate not reported, No ITT analysis, 9 participants from intervention were excluded because they did not use the program.	UC, no protocol to compare with UC	H, baseline characteristics for intervention and control was not reported. Only reported overall	H, randomisation method not reported, participants from intervention group were excluded due to noncompliance
Bostock et al., 2016 [47]^β^	Crossover RCT	L	L	H, unable to blind due to the nature of the study	UC, Not reported	L	UC, protocol mentioned but did not find in public domain	H, possible opportunistic samples	UC, participants self-identifies as having poor sleep were included, not used formal assessment tool i.e DSM-5,
Ebert et al., 2016 [44]	RCT	L	L	H, unable to blind due to the nature of the study	UC, Not reported	L	L	L	L
Grime, 2004 [45]	RCT	L	UC, Not reported	UC	UC, Not reported	UC, no ITT	UC, no protocol	UC, very small sample size (24 in each group),	UC, did not report whether the study allocation were concealed or the outcome assessors were blinded, no protocol
Weber, 2019 et al. [46]	RCT	UC, randomisation method not reported	UC, randomisation method not reported	H, unable to blind due to the nature of the study	UC, Not reported	H, excluded many participants from the app group who didn’t answer the questionnaires which can create bias	H, not enough information on baseline characteristics work productivity outcome was assessed but not reported	H	H, randomisation method not reported, excluded many participants from the app group who didn’t answer the questionnaires
Bolier et al., 2014 & Ketelaar et al., 2013 [48,49]	Cluster RCT	L	L	L	L	L	L	H, possible baseline imbalance: p values for baseline differences between the groups were not reported	UC, possible baseline imbalance: p values for baseline differences between the groups were not reported
Volker et al., 2015 [50]	Cluster RCT	L	L	L	L	L	L	L	L

H = High; L = Low; UC = Unclear

^β^ We used the same assessment for the crossover trial as we only used data of the period prior to crossover

*For the cluster randomised RCTs, the following extra criteria were checked and reported in Column “Other sources of bias”: Recruitment bias (differential participant recruitment in clusters for different interventions); Baseline imbalance; Loss of clusters; Incorrect analysis; Comparability with individually randomized tria

### Physical outcomes

As physical outcomes, no study included in this review published actual measurements of physical conditions, such as cardiovascular disease. However, stress-related physical symptoms related to somatisation and sleep impairment as indicated by study participants were reported.

### Sleep impairment

Three studies reported sleep impairment using the following assessment: Sleep Condition Indicator (SCI) [47], Insomnia Severity Index (ISI) [44], and the Sleeping Troubles Copenhagen Psychosocial Questionnaire-Revised Version (COPSOQ II) [46]. One study which included self-identified “poor” sleep participants, reported statistically significant differences in the intervention group compared to the control at eight weeks [47]. The other study, which included participants with high stress scores, showed significant effect in favour of the intervention at seven weeks and six months [44]. The third study, which measured sleep impairment, did not significantly differ between the control and intervention groups [46].

### Physical health impairment

Two studies reported physical health levels, using self-reported SF 12 and SF 36 v2 [52–54]. Both studies found no statistically significant differences between the intervention and control [44,46].

### Somatisation

One study reported physical symptoms related to somatisation using the Patient Health Questionnaire-15 (PHQ-15) screening tool [55]. There were no statistically significant differences between the eHealth intervention group and the control at one- and three-months post-intervention. But there was a significant improvement of somatisation related physical symptoms in the eHealth intervention group compared to control at 6 and 9 months (p = 0.017 and p = 0.039) [50].

### Psychological outcomes

All seven studies reported psychological outcomes [43–50]. Six studies reported measures of anxiety and depression symptoms, and four studies reported on stress levels, which also included posttraumatic stress [43,44,46,49].

### Anxiety and depression

Six studies measured anxiety and depression outcomes using various assessment tools: the Beck Anxiety Inventory (BAI) [43], the Brief Symptom Inventory (BSI) [48], the Hospital Anxiety and Depression Scale (HADS) [44,45], the Generalised Anxiety Disorder-2(GAD-2) [47], the Patient Health Questionnaire-9 (PHQ-9), the Generalised Anxiety Disorder-7 (GAD-7) [50], and the Centre for Epidemiologic Studies Depression Scale-Revised (CES-D) [43,44]. Out of six, four studies did not find a statistically significant difference between the digital intervention and control condition in anxiety and depression [43,47,48,50].

In contrast, two studies which included employees with higher levels of psychological distress showed significant improvement with digital intervention compared to control [44,45]. The study which worked following the Lazarus and Folkman’s ’Transactional Model of Stress’ [44] included only higher stress level participants in their sample (22 or more on the Perceived Stress Scale (PSS-10)). It showed a significant reduction of anxiety and depression at seven weeks and six months (p<0.001 for both anxiety and depression) [44]. Another study [45] which included only sick listed employees of a UK hospital (4 or more on the General Health Questionnaire 12 (GHQ-12)) showed significantly lower depression scores in the intervention group at the end of the treatment and one-month post-treatment (p = 0.028 and p = 0.04), and lower anxiety scores at one-month post-treatment (p = 0.021), compared to wait-list control. However, at 3- and 6-months post-treatment, these differences were no longer significant.

### Stress

Four studies reported stress outcomes using the different assessment tools (the symptoms of distress scale, the Perceived Stress Score (PSS-10) [56], the Copenhagen Psychosocial Questionnaire-Revised Version (COPSOQ II) [57], the posttraumatic Impact of Event Scale (IES) [58] and the Four Dimensional Symptoms Questionnaire (4DSQ) [43,44,46,49]). Three out of four studies in the intervention group showed significantly lower stress scores than the wait-list control at different time points [43,44,46]. One study showed a significant reduction in stress score at three months (p<0.023) [43] and the other study at seven weeks and six months (p<0.001) [44]. One study which used the JDR model of burnout and measured both general and cognitive stress between the two groups (significant group*time interactions) also showed a significant reduction in stress over time in the intervention group (p<0.001 general stress, p<0.01 cognitive stress) [46]. In contrast, a study [48,49] which included a subgroup analysis of participants with high stress levels, measured both posttraumatic stress and distress did not show significant improvement between the digital intervention group and the control group at six months follow up.

### Wellbeing

Two studies reported employees’ wellbeing using the Warwick- Edinburgh Mental Wellbeing Scale [46] and WHO-5 wellbeing index [48] with mixed results. One study reported that participants in the intervention group showed significantly more wellbeing over time (significant group*time interaction) than the wait-list control group at six weeks follow up [46]. However, the other did not find a statistically significant positive effect with digital intervention on wellbeing at six months [48].

### Other mental-health related outcomes

Three studies reported other mental health-related outcomes such as, resilience, worry and positive mental health, which measured emotional, psychological and social wellbeing [44,46,48,49]. One study found no statistically significant differences between intervention and control in resilience [46]. Two other studies found significant effects in favour of digital intervention in relevant to positive mental health (F = 3.46, p = 0.03, Cohen’s d = 0.37 at three months follow-up, 0.28 at six months follow-up) [48] and employee’s worry and quality of life regarding mental health (p <0.001 at six months) [44]. The results are shown in Table 3.

**Table 3:** Overview of outcomes of the included studies.

First Author, Date, Country	Design	Participants	Mean age (SD) / (Male %)	Intervention and Control	Setting, Intervention programme	Intervention length, delivery modality and support/guidance provided	Measure depression/anxiety/stress at baseline and theoretical underpinning of methods, ITT or PP analysis	Results (Intervention vs. Control) Mean (SD), significance	Summary
**Employees with general levels of psychological distress**									
Billings et al., 2008 [43], USA	RCT	Employees from a technology company in USA	34 (SD not reported), Male (29.4%) mean score, SD PHRQ	Web-based n = 154; wait-list control n = 155	Technology company, CBT based Stress and Mood Management, use of embedded assessment and multimedia elements	3 month web-based, multimedia health promotion program which is tailored to the individual user through baseline assessment, no guidance or support indicated Fu = 3 months	Not measured. No theory PP	1)Anxiety (BAI) 3M: NS 2) Depression(CES-d) 3M: NS 3)Stress (SDS) B: 17.52 (4.53) vs 16.81(3.78) 3M: 16.03(4.18) vs.16.5(4.35), p = 0.023	Stress related measures improve significantly. Anxiety and depression did not improve.
Bolier et al., 2014 & Ketelaar et al., 2013 [48,49], Netherland	Cluster RCT	Nurses and Allied Health Professionals	40 (12), Male (21.2%)	Workers’ health surveillance module (WHS)online intervention n = 178, wait-list no intervention control n = 188	Hospital wards, Range of CBT-based interventions targeting mental fitness; work stress; depressive and panic symptoms and risky drinking behaviour offered following screening	3 month intervention period programmes delivered via website. Feedback provided following screening, access to contact forum provided. Fu = 6 months	Not measured. No theory PP	1) Anxiety (BSI)§ 6M: NS 2) Depression (BSI)§ 6M: NS 3)Stress (IES)§ 6M:NS (subgroup with high stress levels only) 4)Well-being Index (WHO-5)§ 6M: NS 5)Mental Health Continuum- Short Form (MHC-SF)§ B:3.39 (0.66) vs.3.25 (0.74) 6M:3.65 (0.66) vs. 3.33 (0.74), p = 0.03 (cohen’s d = 0.37 at 3 months and 0.28 at 6 months)	No significant improvements in anxiety, depression, and posttraumatic stress. Positive mental health was significantly enhanced in the intervention group, in comparison to the control group
Bostock et al.,✴ 2016 [47], UK	RCT	Employees from a Global ’Fortune 500 ’company who were self-identify as having poor sleep	34(6.01), Male (67%)	digital Cognitive Behavioural Therapy (dCBT) n = 135, wait list control n = 135	Worldwide corporations, CBT based programme is is presented by an animated virtual therapist (‘The Prof’), and tailored by the programme’s algorithms to each individual’s characteristics, personal goals, sleep diary data and progress	8 week digital Cognitive Behavioural Therapy (dCBT) for insomnia was delivered via an established program (www.sleepio.com and associated Sleepio App); No support or human contact. Fu = 8 weeks	Not measured. No theory PP	1)Anxiety 8wk: NS 2) Depression 8wk: NS 3) Sleep Condition Indicator(SCI) B:4.78 (0.14) vs.4.72 (0.14) 8wk: 6.44 (0.16) vs. 6.44 (0.16), Cohen’s d = 1.10 vs 0.34, p<0.0001	No significant improvement for depression or anxiety. Significant improvement for sleep.
Weber et al., 2019 [46], Germany	RCT	Employees from six different businesses in the European countries	41 (11.19), Male (24%)	mHealth n = 210, wait list control n = 322	Various organisations, Kelaa mental Resilience App based on CBT and mindfulness based cognitive therapy	4 weeks intervention (maximum of 28 sessions); personalised feedback on questionnaire scores as well as detailed feedback on sleep data are given within the app. Fu = 6 weeks	Not measured. JDR (job demands-resources model of burnout) PP	1a) Stress, General (COPSOQ II)§ B: 3.00 (0.76) vs. 3.01 (0.73) 6 wk: 2.46 (0.80) vs. 2.57 (0.81), p <0.001 1b) Stress, Cognitive (COPSOQ II)§ B:2.59 (0.85) vs. 2.63 (0.78) 6wk: 2.17 (0.85) vs. 2.34 (0.81), p < 0.01 2) Insomnia (COPSOQ II)§ 6wk: NS 3) Wellbeing(WEMWBS)§ B: 3.26 (0.65) vs. 3.23 (0.60) 6wk: 3.45 (0.78) vs. 3.44 (0.71), p < 0.01	Significant improvement in stress and wellbeing but not insomnia.
**Employees with higher levels of psychological distress**									
**First Author, Date, Country**	**Design**	**Participants**	**Mean age (SD) / (Male %)**	**Intervention and Control**	**Intervention programme**	**Intervention length, delivery modality and support/guidance provided**	**Measure depression/anxiety/stress at baseline and theoretical underpinning of methods, ITT or PP analysis**	**Results (Intervention vs. Control) Mean (SD)**	**Summary**
Ebert et al., 2016 [44], Germany	RCT	Employees from an insurance company with PSS-10 scores ≥22	42 (9), Male(28%)	Internet-based stress management intervention (iSMI) n = 131 or wait list control n = 132	Insurance company, GET.ON Stress’ CBT programme, problem-solving and emotional-regulation strategies	7 week (7 sessions) self-guided programme delivered via website and mobile device. Content was tailored to each participant’s response. No human support. The participants could choose to receive automatic motivational text messages and small exercises on their mobile phones. Fu = 6 months	Only included participants with scores ≥22 on the Perceived Stress Scale (PSS-10). Lazarus and Folkman ’ Transactional Model of Stress’ ITT	1)Anxiety (HADS-A) B;11.4(3.4) vs. 11.3(3.6) 7wk: 8.0(3.7) vs 9.9(3.8), p<0.001 6M: 7.2(3.7) vs. 9.3(4.2), p<0.001 2) Dépression(CES-D) B: 25.1(9.31) vs. 23.9(8.3) 7wk: 16.1(8.7) vs 21.4(9.1), p<0.001 6M: 15.2(9.0) vs 20.2(10.0), p<0.001 3) Stress (PSS-10) B: 25.7 (5.0) vs. 26.1 (4.1) 7wk: 18.1(5.7) vs. 23.4(5.4), p<0.001 6M: 17.5(6.7) vs. 21.8(6.7), p<0.001 4) Insomnia Severity(ISI) B: 13.0(5.6) vs. 12.8(6.0) 7wk: 9.3(5.2) vs. 11.2(6.5), p<0.001 6M: 8.0(5.1) vs. 10.3(6.0), p<0.001	Improvement of sleep, anxiety, depression and stress
Volker et al.,✴ 2015 [50], Netherland	Cluster RCT	Sick-listed employees with common mental disorders who were on sickness absence between 4 and 26 weeks and screened positive (score ≥10) on either PHQ-9 and/or PHQ-15 and/or GAD-7	45 (10), Male (40%)	E-health module embedded in Collaborative Occupational health care (ECO)n = 131, Care as usual n = 89	Various companies, Return@Work’ Pyscho-education, CBT, problem-solving, pain/fatigue management and relapse prevention.	5 modules (up to 16 sessions, tailored to individual) over 3 months combined with occupational physician consultations who received automated email that were based on decision aid. Fu = 12 months	Only included participants who screened positive (score ≥10) on either the depression scale of the PHQ-9 and/or the somatization scale of the PHQ-15 and/or the GAD-7. No theory ITT	1)Anxiety (GAD7) 3M, 6M, 9M, 12M: NS 2) Depression (PHQ9) 3M, 6M, 9M, 12M: NS 3)Somatisation (PHQ15) B:12.54(4.3) vs. 13.03 (4.9) 9M: 8.45 (5.1) vs. 10.11 (4.9), p = 0.017 12M:8.01 (5.04) vs. 9.47 (5.2), p = 0.039 3M and 6M: NS	No significant difference in depression and anxiety between intervention and control. But significant improvement in stress/somatisation related physical symptoms at 9 and 12 months
Grime, 2004 [45], UK	RCT	Employees from the London NHS occupational health department who were on sick leave for 10 or more cumulative days due to stress, anxiety or depression in the past 6 months, and scored ≥ 4 on GHQ-12	39 (9), Male (42%)	‘Beating The Blues’ plus conventional care n = 24, conventional care n = 24	London NHS occupational health department, Beating The Blues’ computerised CBT programme aims to challenge specific thinking patterns and implement behavioural change. It concludes with a therapy map or programme review, goal setting and action planning.	8 weeks (8 sessions) CBT programme was loaded onto a standalone computer in a private room in the occupational Health Department.; all participants received conventional care. Fu = 8 weeks	Only included participants who scored 4 or more on the GHQ-12 (General Health Questionnaire). No theory PP	1)Anxiety (HADS) B: 11.75(3.87) vs. 14.04 (4.34), 1M:8.20(3.95) vs. 12.00 (3.61), p = 0.021 End of treatment, 3M and 6 M: NS 2) Depression (HADS-D) B: 7.96 (3.43) vs. 10.63 (4.13) End of treatment:: 5.38 (3.93) vs. 8.61 (3.86), p = 0.028 1 M: 5.00 (3.32) vs. 8.53 (3.82), p = 0.040 3M and 6M:NS	Significant improvement in depression and anxiety at 1 month after the end of treatment but not 3 and 6 months follow up.

✴results were provided by the study authors, § = group*time interaction. B = baseline, Fu = follow up, NS = Non-significant, ITT = Intention to treat, PP = Per protocol Only significant differences are shown.

SDS = Symptoms of distress scale BAI = Beck Anxiety Inventory CES-D = Center for Epidemiological Studies BSI = Brief Symptom Inventory ISI = Insomnia Severity Index HADS-A = Hospital Anxiety and Depression Scale

PSS-10 = Perceived Stress Scale GAD-2 = Generalised Anxiety Disorder-2 item, COPSOQ II = Copenhagen Psychosocial Questionnaire–Revised Version(general stress) WEMWBS = Warwick-Edinburgh Mental Wellbeing Scale WHO-5 = WHO Well-being Index IES = Impact of Event Scale SCI = Sleep Condition Indicator PHQ-9 = Patient Health Questionnaire-9 PHQ-15 = Patient Health Questionnaire-15

GAD-7 = Generalised Anxiety Disorder-

### Work-related outcomes

Seven publications (six studies) reported work-related outcomes [43,44,46–50] regarding presenteeism and absenteeism in several ways and using several instruments, which is summarised in Table 4 below. Only significant findings are shown.

**Table 4:** Work-related outcomes.

Study	Presenteeism Instrument	Presenteeism result	Absenteeism Instrument	Absenteeism result	Summary
Billings et al., 2008 [43]	Work productivity	NS at 3 months			
Bolier et al., 2014 [48],Ketelaar et al., 2013 [49]	UWES work engagement Work Ability Index Nurses Work Functioning Questionnaire (NWFQ)	F = 3.44, p = 0.03, Cohen’s d = 0.25 at 3 months, 0.15 at 6 months. NS at 6 months NS for subgroup analysis of participants with high stress levels Statistically significant group*time interaction effect (P = 0.4) for all participants at 6 months.			Small positive effect on presenteeism in the intervention group in terms of work engagement, but NS on Work Ability Index
Bostock et al., 2016 [47]	WPAI presenteeism scale	On the WPAI presenteeism scale, a 15.4% reduction in reports of poor sleep affecting productivity at work was observed following digital CBT(2.4% following WL), representing a significant [F = 10.99, P = 0.001], and medium effect in terms of Cohen criteria (d = 0.67)(change from baseline at 8 weeks post-treatment). There was no significant change in the control group.	WPAI absenteeism scale	On the WPAI absenteeism scale, a small effect was associated with pre-post change after digital CBT (d = 0.32), versus minimal effects after WL, but the interaction term was not significant (F = 2.70, P = 0.101). NS for absenteeism at 8 weeks	Significant positive effect in terms of presenteeism, NS for absenteeism
Ebert et al., 2016 [44]	UWES Presenteeism (TIC-P-G)	F = 5.4, p<0.05, Cohen’s d = 0.17 (95% CI -0.08 to 0.41) at 7 weeks NS at 6 months F = 9.3, p<0.01, d = 0.30, (95% CI 0.06 to 0.54) at 6 months	Absenteeism (TiC–P-G)	NS at 6 months	Significant improvement of presenteeism s per UWES in favour of the intervention as short term, not long-term outcome. At 6 -month follow-up, presenteeism significantly improved as per TIC-P in the experimental group (p<0.01) No significant difference in absenteeism between the two groups.
Volker et al., 2015 [50]			(TiC–P)combined with social insurance data	The median duration was 77.0 (IQR 29.0–152.3) days in the control group and 50.0 (IQR 20.8–99.0) days in the E-health group, a difference of 27 days (hazard ratio [HR] 1.390, 95% CI 1.034–1.870, P = .03) for first RTW at 12 months NS for full RTW at 12 months	The duration until first RTW improved in the intervention and differed significantly between the groups. No significant difference was found for full RTW
Weber et al., 2019 [46]	WPAI	Authors collected the data but did not analyse			Authors did not analyse data for WPAI

TiC–P-G = Medical Technology Assessment Cost Questionnaire for Psychiatry WPAI = Work Productivity and Activity Impairment

WAI = work functioning and Work Ability Index UWES = Utrecht Work Engagement Scale RTW = Return to work

CBT = Cognitive Behavioural Therapy NS = non-significant

### Presenteeism

Five studies reported some form of work performance that in this review were taken as an expression of presenteeism, which illustrates that the definition and operationalisation of presenteeism is not comparable between the instruments and consequently studies. Some studies used two instruments [44,48,49]. Two studies using the Utrecht Work Engagement Scale (UWES) [44,48] reported a small positive effect on work engagement in the intervention group (F = 3.44, p = 0.03, Cohen’s d = 0.25 three months follow-up, 0.15 six months follow-up) [48] and a statistically significant difference between groups in favour of the intervention at the seven week follow up (F = 5.4, p<0.05, Cohen’s d = 0.17) but not at six months [44]. One study [49] used an item of the Work Ability Index in which an individual assesses their own workability by comparing their actual one with the highest ever; and another instrument, the Nurses Work Functioning Questionnaire (NWFQ). This consists of 7 subscales: (1) cognitive aspects of task execution and general incidents, (2) impaired decision making, (3) causing incidents at work, (4) avoidance behaviour, (5) conflicts and annoyances with colleagues, (6) impaired contact with patients and their family and (7) lack of energy and motivation. This study did not find any significant differences between the intervention and control group [49]. Three other studies collected the data for presenteeism using the WPAI and the Work Limitations Questionnaire [43,46]; one did not analyse the data [46], one found a statistically significant difference in presenteeism (p = 0.001) [47] and the other found no significant difference in work productivity between the web-based intervention and wait-list control at three months [43]. One study that assessed presenteeism with a dedicated instrument at 6 months (TiC-P) [44] found a statistically significant improvement in presenteeism (p<0.01).

### Absenteeism

One study used the Treatment Inventory Cost in Psychiatric Patients (TIC-P) combined with social insurance data to assess Return to Work (RTW) on sick-listed employees with common mental disorders. They reported absenteeism as duration until first return to work and complete return to work after a long-term sickness. The duration until the first RTW in the intervention group was significantly shorter than in the control group (P = .03). The median duration was 77.0 (IQR 29.0–152.3) days in the control group and 50.0 (IQR 20.8–99.0) days in the intervention group, a difference of 27 days (hazard ratio [HR] 1.390, 95% CI 1.034–1.870, P = .03) at 12 months [50]. Two studies assessing absenteeism with a dedicated instrument at 8 weeks (WPAI) [47] and 6 months (TiC-P) [44] found no statistically significant difference.

### Adherence and uptake

In terms of adherence and uptake to the digital intervention, all studies mentioned some kind of adherence level. Five studies reported that participants in the digital intervention groups logged in to the intervention materials at least one time (range 16% to 82%) [43,44,46,48–50]. Two studies reported participants in the intervention group either attended or completed four or more sessions of the study materials [45,47]. Four studies [43,44,47,50] reported good uptake, one of them with partly moderate uptake of the future planning/booster parts [44]. One study [48,49] reported low uptake, and one study moderate uptake [46]. Follow up adherence was unknown in two studies [43,44], very low in one study [48,49], good in one study [47], very good in one [45], and moderate in two [46,50]. In general, uptake was better than adherence. Of all studies, only two had both good uptake and moderate to good adherence [47,50]. The findings regarding adherence and uptake are shown in Table 5.

**Table 5:** Adherence and Uptake.

Authors	Excluded due to lack of adherence	Logged in at least once	Logged in 1 or more times	Attended 4 or more sessions	Summary
Billings et al., 2008 [43]	9 (5.8%)	Out of 154 participants, - 100 (65%) reviewed Stress management, - 122 (79%) reviewed Depression, - 131 (85%) reviewed Anxiety and - 134 (87%) reviewed Treatment material			Good uptake (65–87%), Follow up adherence unknown
Bolier et al., 2014 & Ketelaar et al., 2013 [48,49]		Out of 178, 28(16%) of all participants logged in at least once. - 22 (12%) logged into Psyfit, - 4 (2%) Colour your Life - 7 (4%) Strong at work. None logged into Don’t Panic Online or Drinking Less. 6 (8%) out of a subgroup of 17 (23%)) who had high stress levels, logged into Psyfit and 3 (4%) logged into Colour your Life	Out of 178, 28(16%) participants logged in at least once. 9(5%) started one or more modules		Low uptake (0 to 12% for all participants and 4 to-23% for subgroup with high stress levels), Follow up adherence very low
Bostock et al., 2016 [47]		81% participants recorded sleep diaries for two weeks (or more), 67% for three weeks, 47% for four weeks and 32% for six weeks or more		63 (47%) attended four or more sessions	Between moderate and good uptake (32% to 81%) Good adherence (47%)
Ebert et al., 2016 [44]		Out of 131 participants, - 113 (82%) logged into Psycho-education, - 97 (74%) to PS I–Learning phase - 87 (66%) to PS II–Maintenance phase, - 80 (61%) to ER I–Muscle- & breathing relaxation, - 74 (56%) to ER II–Acceptance and tolerance of emotions, - 65 (50%) to ER III–Effective self-support in difficult situations, - 55 (42%) to Plan for the future and - 37 (28%) to Booster session			Good uptake for psycho-education, problem solving, emotion regulation (50–82%), Moderate uptake for future planning or booster sessions (28–42%) Follow up adherence unknown
Grime, 2004 [45]			5(21%) completed 2–3 sessions	16(67%) completed all eight sessions	Good adherence (67%)
Volker et al., 2015 [50]		100 Out of 131 (76%) logged in to the intervention.	From those 100 participants who logged in, 10% (10/100) didn’t finish the intro but 90% (90/100) finished the intro and started return@work modules.	36 out of 90 (40%) in the group that logged in completed half of the modules of return@work.	Good uptake (76%) Moderate adherence (40%)
Weber et al., 2019 [46]	137/347 (39.5%) from the App group were excluded. 9/331 (3%) who downloaded App were excluded in the control group.	111 (52.9%) participants in the App group did not track their sleep	22(10.5%) only tried once to track their sleep	On average 11.06 out of 28 sessions were completed in the App group and an average 3.61 out of 28 nights were tracked.	Uptake moderate on tracking sleep (47.1%) Moderate adherence

### Attrition rates

Six out of seven studies reported dropout rates for follow up which were calculated based on numbers at baseline compared with those included in the final analysis for each study arm [44–50]. The employees in the intervention arm were more likely to drop out compared to the control arm (drop out ranged from 17% to 61% in the intervention arm and 0% to 36% in the control arm).

## Discussion

### Summary of the main results

This review of seven randomised controlled trials evaluated tailored digital interventions versus waiting list control or usual guidance to improve stress in the workplace yielded mixed results.

The study outcomes were positive in terms of presenteeism, sleep, stress levels and physical symptoms related to somatisation. However, they are less so for addressing depression and anxiety and absenteeism.

At first glance, this seems understandable, as the interventions addressed work stress, which is associated with presenteeism. However, this does not automatically lead to improvement in more serious conditions such as depression, anxiety, or sickness absence.

At second glance, although tailored digital interventions did not reduce anxiety and depression in the general working population, they significantly reduced depression and anxiety in employees with higher levels of psychological distress. Moreover, most studies did not show improvement in absenteeism, they did show faster return to work in employees on long-term sick leave who received a tailored intervention. This suggests that tailored digital interventions can be of use for preventive approaches for stress, sleep, presenteeism and somatisation in the workplace, and for more serious depressive and anxiety symptoms and absenteeism. General preventive measures work well to address work stress. But relieving work stress in itself does not reduce depressive or anxiety disorders per se. For that, a tailored approach is needed, that focuses on treatment of depression and anxiety and this is provided in the group with serious depressive and anxiety disorders. So this is a benefit of the tailoring of the intervention.

The literature about interventions to address mental disorders in the workplace shows that treatment for mental disorders does not improve work functioning automatically. For that, separate interventions are needed that address work functioning. This can be achieved by triage and tailoring. Given the results, tailoring seems to be an approach that can bear fruit [59–62]. Technology and clinical practice can complement each other in blended eHealth. For example, the blended study [50] found improved physical symptoms suggesting somatisation and improved first return to work. This study provided the employees with self-help modules and monitored progress based upon their self-reported outcomes. It gave the occupational physician guiding the employee to return to work tailored suggestions to improve guidance and refer for treatment of medical conditions or psychological symptoms if needed. This is an example of a blended digital intervention that may have particular benefits in employees with more severe symptoms and absenteeism.

An advantage of digital interventions for employees in psychological distress may be that participants remain anonymous by delivering the interventions through the internet [63]. This has the potential to reduce stigma associated with addressing mental health problems. The intervention can also be accessible at any time and place and is easily scalable [16,31]. These characteristics enhance the potential of reaching a greater proportion of the eligible population while being cost effective as only a small increase of resources is required [30].

Our systematic literature review indicates presenteeism seems to be a significant outcome for digital tailored interventions in the workplace. However, the definition and operationalisation using different instrument for measurement of presenteeism varies. Hence, transparency and harmonization of the definition and operationalisation of presenteeism in (future) research is needed [64]. Moreover, digital interventions at the workplace are often subject of cost-effectiveness analyses.

### Digital intervention tailoring and personalisation

One thing that sets this systematic review apart from earlier ones is the focus on digital interventions which offer tailoring for participants. Personalisation is essential to developing a tailored digital intervention and may influence its effectiveness. The type of personalisation and tailoring offered by a digital intervention and how it targets mental health symptoms impacts the user experience. Traditional in-person treatment is highly personalised, based on clinician and patient interaction. Interventions or techniques can be changed by the clinician at any time, allowing flexibility in care. While digital interventions can never be equivalent, there is the possibility for them to provide effective targeted support, either alongside in-person treatment (blended treatment) or as an alternative [65].

### Adherence

Apart from one study [48,49], most studies had moderate or good uptake whereas follow up adherence varied. It was not reported in two studies [43,44], and in the remainder of studies ranged from very low to very good. The general adherence was better than in general in non-tailored digital programs, and it is possible that this may have to do with the tailoring provided as tailoring makes it worthwhile for the employee to engage with the digital intervention repeatedly [66].

### Strengths and limitations

The primary strength of this study is that we included data without language restriction from studies identified by a comprehensive search of the published literature. We included all studies exploring the effect of both physical and mental components outcomes. As there have been no reviews on tailored digital interventions for employees with work-related stress before, this systematic review is innovative.

Even though this review found improvements in various symptoms and work productivity as a result of the digital intervention, there are several limitations. Firstly, we only searched google scholar for grey literatures and there was a possibility that some studies could have been missed due to double screening for only 10% of title and abstract [67,68]. Secondly, caution is advised when interpreting the results, as only two of the seven studies were of high quality and there was a variety of outcome measures. Thirdly, the results of most of the studies relied exclusively on self-report measures over a relatively short term, except for one study [50]. There was one study that we could not report on as shown in the flowchart, as despite our efforts to contact the author, we could not include that study due to a lack of response. *Fourthly*, there was considerable heterogeneity in assessment tools, outcomes, components of digital interventions, and time points, making meta-analysis impossible to perform. Especially, in the seven studies evaluating work productivity, six different instruments were used, most of them not validated. Finally, many of the included studies were conducted in high-income countries; therefore, our findings may not be generalisable to low-income countries.

## Recommendations for research

Given the promising results, further research into tailored digital interventions to address work stress is needed. Also, tailor interventions should follow the reporting standards recommended by Harrington and Noar (2012) [69]. Given the scarcity of digital interventions evaluated by research so far, this should be a research priority and including blended aspects in such interventions may be beneficial. It would be recommended to use validated questionnaires to assess work-productivity i.e., TiC-P [70]. Furthermore, future studies should investigate the durability of these effects over longer periods of time. Studies also should report on adherence to the intervention, both in terms of how often employees log in, and whether they finish the intervention. Moreover, more broad reflection on factors affecting the effectiveness of such intervention is needed. It should include such phenomena as individual motivation to solve their own problems, organisational culture and type of work performed, educational level, attitudes towards mental health issues, stigma and many others.

## Conclusions

This review shows that tailored digital interventions have promising results in terms of presenteeism, stress levels, sleep and physical symptoms related to somatisation but less so for absenteeism. They did not reduce anxiety and depression in the general working population; however, they can significantly reduce depression and anxiety in employees with higher levels of psychological distress. There is a need for uniformity in the use of assessment tools, outcomes, and proper reporting of components of digital interventions in research in this domain, especially regarding work productivity.

## Supporting information

S1 Appendix Full search strategy. (ZIP)

S2 Appendix Tailored interventions table. (DOCX)

S3 Appendix EMPOWER PRISMA checklist. (DOC)
