# Supplementary material for: Effectiveness of tailored digital health interventions for mental health at the workplace: A systematic review of randomised controlled trials
Source: PLOS Digit Health. 2022 Oct 21;1(10):e0000123. doi: 10.1371/journal.pdig.0000123 (PMC9931277; doi:10.1371/journal.pdig.0000123)
Supplement: S1 Appendix — (ZIP) [file pdig.0000123.s001.zip › EMPOWER Search Strategy Final/EMPOWER EMBASE FINAL.docx]

**EMPOWER EMBASE**

1. depression/ or major depression/ or anxiety disorder/

2. life stress/ or physical stress/ or environmental stress/ or mental stress/ or physiological stress/ or stress/ or job stress/

3. physiological stress/

4. professional burnout/

5. 1 or 2 or 3 or 4

6. exp computer/

7. mobile phone/ or smartphone/

8. text messaging/

9. Internet/

10. wearable computer/

11. e-mail/

12. computer assisted therapy/

13. 6 or 7 or 8 or 9 or 10 or 11 or 12

14. cognitive behavioral therapy/

15. self care/

16. problem solving/

17. mindfulness/

18. counseling/

19. psychotherapy/

20. psychiatry/

21. health promotion/

22. 14 or 15 or 16 or 17 or 18 or 19 or 20 or 21

23. 5 and 13 and 22

24. ((digital or decision aid$ or ehealth or e-health or ihealth or i-health or mhealth or m-health or online or on-line or internet-based or internet$ or web-based or web$ or e-mail) adj3 (stress or burnout or burn-out)).ti,ab.

25. ((digital or decision aid$ or ehealth or e-health or ihealth or i-health or mhealth or m-health or online or on-line or internet-based or internet$ or web-based or web$ or e-mail) adj3 (problem solv$ or problem-solv$)).ti,ab.

26. ((digital or decision aid$ or ehealth or e-health or ihealth or i-health or mhealth or m-health or online or on-line or internet-based or internet$ or web-based or web$ or email) adj3 (self help or self-help or selfhelp or self care or self-care or self care)).ti,ab.

27. ((digital or decision aid$ or ehealth or e-health or ihealth or i-health or mhealth or m-health or online or on-line or internet-based or internet$ or web-based or web$ or e-mail) adj3 (CBT or cognitive therap$ or cognitive behav$ therap$)).ti,ab.

28. (iCBT or icognitive or i-cognitive).ti,ab.

29. ((digital or decision aid$ or ehealth or e-health or ihealth or i-health or mhealth or m-health or online or on-line or internet-based or internet$ or web-based or web$ or email) adj3 mindfulness$).ti,ab.

30. ((digital or decision aid$ or ehealth or e-health or ihealth or i-health or mhealth or m-health or online or on-line or internet-based or internet$ or web-based or web$ or e-mail) adj3 (psychotherap$ or psychiatr$ or counsel$)).ti,ab.

31. ((digital or decision aid$ or ehealth or e-health or ihealth or i-health or mhealth or m-health or online or on-line or internet-based or internet$ or web-based or web$ or e-mail) adj3 (depress$ or anxiety or anxious)).ti,ab.

32. ((digital or decision aid$ or ehealth or e-health or ihealth or i-health or mhealth or m-health or online or on-line or internet-based or internet$ or web-based or web$ or e-mail) adj3 health promot$).ti,ab.

33. ((smartphone$ or smart-phone$ or smart phone$ or cellphone$ or cell-phone$ or cell phone$ or mobile phone$ or android$ or iPhone$ or sms messag$ or text messag$ or texting or computer$ or app or apps or (application$ adj3 phone$)) adj3 (stress or burnout or burn-out)).ti,ab.

34. ((smartphone$ or smart-phone$ or smart phone$ or cellphone$ or cell-phone$ or cell phone$ or mobile phone$ or android$ or iPhone$ or sms messag$ or text messag$ or texting or computer$ or app or apps or (application$ adj3 phone$)) adj3 (problem solv$ or problem-solv$)).ti,ab.

35. ((smartphone$ or smart-phone$ or smart phone$ or cellphone$ or cell-phone$ or cell phone$ or mobile phone$ or android$ or iPhone$ or sms messag$ or text messag$ or texting or computer$ or app or apps or (application$ adj3 phone$)) adj3 (self help or self-help or selfhelp or selfcare or self-care or self care)).ti,ab.

36. ((smartphone$ or smart-phone$ or smart phone$ or cellphone$ or cell-phone$ or cell phone$ or mobile phone$ or android$ or iPhone$ or sms messag$ or text messag$ or texting or computer$ or app or apps or (application$ adj3 phone$)) adj3 (CBT or cognitive therap$ or cognitive behav$ therap$)).ti,ab.

37. ((smartphone$ or smart-phone$ or smart phone$ or cellphone$ or cell-phone$ or cell phone$ or mobile phone$ or android$ or iPhone$ or sms messag$ or text messag$ or texting or computer$ or app or apps or (application$ adj3 phone$)) adj3 mindfulness).ti,ab.

38. ((smartphone$ or smart-phone$ or smart phone$ or cellphone$ or cell-phone$ or cell phone$ or mobile phone$ or android$ or iPhone$ or sms messag$ or text messag$ or texting or computer$ or app or apps or (application$ adj3 phone$)) adj3 (psychotherap$ or psychiatr$ or counsel$)).ti,ab.

39. ((smartphone$ or smart-phone$ or smart phone$ or cellphone$ or cell-phone$ or cell phone$ or mobile phone$ or android$ or iPhone$ or sms messag$ or text messag$ or texting or computer$ or app or apps or (application$ adj3 phone$)) adj3 (depress$ or anxiety or anxious)).ti,ab.

40. ((smartphone$ or smart-phone$ or smart phone$ or cellphone$ or cell-phone$ or cell phone$ or mobile phone$ or android$ or iPhone$ or sms messag$ or text messag$ or texting or computer$ or app or apps or (application$ adj3 phone$)) adj3 health promot$).ti,ab.

41. (wearable adj3 (device$ or technolog$) adj3 (stress or burnout or burn-out)).ti,ab.

42. (wearables adj3 (problem solv$ or problem-solv$)).ti,ab.

43. (wearable adj3 (device$ or technolog$) adj3 (problem solv$ or problem-solv$)).ti,ab.

44. (wearables$ adj3 (problem solv$ or problem-solv$)).ti,ab.

45. (wearable adj3 (device$ or technolog$) adj3 (self help or self-help or selfhelp or selfcare or self-care or self care)).ti,ab.

46. (wearables adj3 (self help or self-help or selfhelp)).ti,ab.

47. (wearable adj3 (device$ or technolog$) adj3 (CBT or cognitive therap$ or cognitive behav$ therap$)).ti,ab.

48. (wearables adj3 (CBT or cognitive therap$ or cognitive behav$ therap$)).ti,ab.

49. (wearable adj3 (device$ or technolog$) adj3 mindfulness).ti,ab.

50. (wearables adj3 mindfulness).ti,ab.

51. (wearable adj3 (device$ or technolog$) adj3 (psychotherap$ or psychiatr$ or counsel$)).ti,ab.

52. (wearable adj3 (device$ or technolog$) adj3 (depress$ or anxiety or anxious)).ti,ab.

53. (wearables adj3 (psychotherap$ or psychiatr$ or counsel$)).ti,ab.

54. (wearables adj3 health promot$).ti,ab.

55. ((digital or decision aid$ or ehealth or e-health or ihealth or i-health or mhealth or m-health or online or on-line or internet-based or internet$ or web-based or web$ or email) adj3 (well-being or wellbeing or resilience or stress$ or mental health or depress$ or anxiety or anxious)).ti,ab.

56. ((smartphone$ or smart-phone$ or smart phone$ or cellphone$ or cell-phone$ or cell phone$ or mobile phone$ or android$ or iPhone$ or sms messag$ or text messag$ or texting or computer$ or app or apps or (application$ adj3 phone$)) adj3 (well-being or wellbeing or resilience or stress$ or mental health or depress$ or anxiety or anxious)).ti,ab.

57. (wearable adj3 (device$ or technolog$) adj3 (well-being or wellbeing or resilience or stress$ or mental health or depress$ or anxiety or anxious)).ti,ab.

58. 24 or 25 or 26 or 27 or 28 or 29 or 30 or 31 or 32 or 33 or 34 or 35 or 36 or 37 or 38 or 39 or 40 or 41 or 42 or 43 or 44 or 45 or 46 or 47 or 48 or 49 or 50 or 51 or 52 or 53 or 54 or 55 or 56 or 57

59. 23 or 58

60. return to work/ or work/ or work engagement/

61. employment/

62. absenteeism/ or presenteeism/ or productivity/

63. medical leave/

64. occupational health/ or occupational therapist/

65. occupational health service/

66. (employee$ or worker$).ti,ab.

67. (employment or occupation or work or workplace$ or worksite$).ti,ab.

68. (burn-out or burnout or work engagement or work-engagement or absenteeism or presenteeism).ti,ab.

69. (return-to-work or return to work).ti,ab.

70. (sick$ adj1 (absence or absent or leave or listed)).ti,ab.

71. 60 or 61 or 62 or 63 or 64 or 65 or 66 or 67 or 68 or 69 or 70

72. 59 and 71

73. (e-mental health or EMH).ti,ab.

74. 71 and 73

75. ((digital or decision aid$ or ehealth or e-health or mhealth or m-health or online or on-line or internet-based or internet* or web-based or web$) adj3 (intervention$ or treatment$)).ti,ab.

76. ((smartphone$ or smart-phone$ or smart phone$ or cellphone$ or cell-phone$ or cell phone$ or mobile phone$ or android$ or iPhone$ or sms messag$ or text messag$ or texting or computer$ or app or apps or (application$ adj3 phone$)) adj3 (intervention$ or treatment$)).ti,ab.

77. (wearable adj3 (device$ or technolog$) adj3 (intervention$ or treatment$)).ti,ab.

78. (wearables adj3 (intervention or treatment$)).ti,ab.

79. (75 or 76 or 77 or 78) and 71 and 5

80. ((digital or decision aid$ or ehealth or e-health or mhealth or m-health or online or on-line or internet-based or internet* or web-based or web$) adj3 (train$ or program$)).ti,ab.

81. ((smartphone$ or smart-phone$ or smart phone$ or cellphone$ or cell-phone$ or cell phone$ or mobile phone$ or android$ or iPhone$ or sms messag$ or text messag$ or texting or computer$ or app or apps or (application$ adj3 phone$)) adj3 (train$ or program$)).ti,ab.

82. (wearable adj3 (device$ or technolog$) adj3 (train$ or program$)).ti,ab.

83. (wearables adj3 (train$ or program$)).ti,ab.

84. (80 or 81 or 82 or 83) and 71 and 5

85. 72 or 74 or 79 or 84

86. randomized controlled trial/

87. Controlled clinical trial/

88. random$.ti,ab.

89. randomization/

90. intermethod comparison/

91. placebo.ti,ab.

92. (compare or compared or comparison).ti.

93. ((evaluated or evaluate or evaluating or assessed or assess) and (compare or compared or comparing or comparison)).ab.

94. (open adj label).ti,ab.

95. ((double or single or doubly or singly) adj (blind or blinded or blindly)).ti,ab.

96. double blind procedure/

97. parallel group$1.ti,ab.

98. (crossover or cross over).ti,ab.

99. ((assign$ or match or matched or allocation) adj5 (alternate or group$1 or intervention$1 or patient$1 or subject$1 or participant$1)).ti,ab.

100. (assigned or allocated).ti,ab.

101. (controlled adj7 (study or design or trial)).ti,ab.

102. (volunteer or volunteers).ti,ab.

103. human experiment/

104. (stepped wedge or (cluster adj2 random$)).ti,ab.

105. trial.ti.

106. 86 or 87 or 88 or 89 or 90 or 91 or 92 or 93 or 94 or 95 or 96 or 97 or 98 or 99 or 100 or 101 or 102 or 103 or 104 or 105

107. (animal/ or animal experiment/ or animal model/ or animal tissue/ or nonhuman/) not exp human/

108. editorial.pt. or case report.ti.

109. 106 not (107 or 108)

110. 85 and 109
