# Supplementary material for: Effectiveness of tailored digital health interventions for mental health at the workplace: A systematic review of randomised controlled trials
Source: PLOS Digit Health. 2022 Oct 21;1(10):e0000123. doi: 10.1371/journal.pdig.0000123 (PMC9931277; doi:10.1371/journal.pdig.0000123)
Supplement: S1 Appendix — (ZIP) [file pdig.0000123.s001.zip › EMPOWER Search Strategy Final/EMPOWER Psycinfo FINAL.docx]

**EMPOWER Psycinfo**

Psycinfo DIFFERENT MESH

1. exp Mental Disorders/

2. exp Anxiety Disorders/

3. exp Major Depression/ or exp Affective Disorders/

4. exp "Depression (Emotion)"/

5. exp Psychological Stress/

6. exp Physiological Stress/

7. exp Occupational Stress/

8. 1 or 2 or 3 or 4 or 5 or 6 or 7

9. exp Computers/

10. exp Smartphones/

11. exp Mobile Phones/

12. exp Text Messaging/

13. exp Internet/

14. exp Wearable Devices/

15. exp Computer Assisted Therapy/

16. 9 or 10 or 11 or 12 or 13 or 14 or 15

17. exp Cognitive Behavior Therapy/

18. exp Self-Care/

19. exp Problem Solving/

20. exp Mindfulness-Based Interventions/ or exp Mindfulness/

21. exp Counseling/

22. exp Psychotherapy/

23. exp Psychiatry/

24. exp Health Promotion/

25. 17 or 18 or 19 or 20 or 21 or 22 or 23 or 24

26. 8 and 16 and 25

27. ((digital or decision aid$ or ehealth or e-health or ihealth or i-health or mhealth or m-health or online or on-line or internet-based or internet$ or web-based or web$ or e-mail) adj3 (stress or burnout or burn-out)).ti,ab.

28. ((digital or decision aid$ or ehealth or e-health or ihealth or i-health or mhealth or m-health or online or on-line or internet-based or internet$ or web-based or web$ or e-mail) adj3 (problem solv$ or problem-solv$)).ti,ab.

29. ((digital or decision aid$ or ehealth or e-health or ihealth or i-health or mhealth or m-health or online or on-line or internet-based or internet$ or web-based or web$ or email) adj3 (self help or self-help or selfhelp or self care or self-care or self care)).ti,ab.

30. ((digital or decision aid$ or ehealth or e-health or ihealth or i-health or mhealth or m-health or online or on-line or internet-based or internet$ or web-based or web$ or e-mail) adj3 (CBT or cognitive therap$ or cognitive behav$ therap$)).ti,ab.

31. (iCBT or icognitive or i-cognitive).ti,ab.

32. ((digital or decision aid$ or ehealth or e-health or ihealth or i-health or mhealth or m-health or online or on-line or internet-based or internet$ or web-based or web$ or email) adj3 mindfulness$).ti,ab.

33. ((digital or decision aid$ or ehealth or e-health or ihealth or i-health or mhealth or m-health or online or on-line or internet-based or internet$ or web-based or web$ or e-mail) adj3 (psychotherap$ or psychiatr$ or counsel$)).ti,ab.

34. ((digital or decision aid$ or ehealth or e-health or ihealth or i-health or mhealth or m-health or online or on-line or internet-based or internet$ or web-based or web$ or e-mail) adj3 (depress$ or anxiety or anxious)).ti,ab.

35. ((digital or decision aid$ or ehealth or e-health or ihealth or i-health or mhealth or m-health or online or on-line or internet-based or internet$ or web-based or web$ or e-mail) adj3 health promot$).ti,ab.

36. ((smartphone$ or smart-phone$ or smart phone$ or cellphone$ or cell-phone$ or cell phone$ or mobile phone$ or android$ or iPhone$ or sms messag$ or text messag$ or texting or computer$ or app or apps or (application$ adj3 phone$)) adj3 (stress or burnout or burn-out)).ti,ab.

37. ((smartphone$ or smart-phone$ or smart phone$ or cellphone$ or cell-phone$ or cell phone$ or mobile phone$ or android$ or iPhone$ or sms messag$ or text messag$ or texting or computer$ or app or apps or (application$ adj3 phone$)) adj3 (problem solv$ or problem-solv$)).ti,ab.

38. ((smartphone$ or smart-phone$ or smart phone$ or cellphone$ or cell-phone$ or cell phone$ or mobile phone$ or android$ or iPhone$ or sms messag$ or text messag$ or texting or computer$ or app or apps or (application$ adj3 phone$)) adj3 (self help or self-help or selfhelp or selfcare or self-care or self care)).ti,ab.

39. ((smartphone$ or smart-phone$ or smart phone$ or cellphone$ or cell-phone$ or cell phone$ or mobile phone$ or android$ or iPhone$ or sms messag$ or text messag$ or texting or computer$ or app or apps or (application$ adj3 phone$)) adj3 (CBT or cognitive therap$ or cognitive behav$ therap$)).ti,ab.

40. ((smartphone$ or smart-phone$ or smart phone$ or cellphone$ or cell-phone$ or cell phone$ or mobile phone$ or android$ or iPhone$ or sms messag$ or text messag$ or texting or computer$ or app or apps or (application$ adj3 phone$)) adj3 mindfulness).ti,ab.

41. ((smartphone$ or smart-phone$ or smart phone$ or cellphone$ or cell-phone$ or cell phone$ or mobile phone$ or android$ or iPhone$ or sms messag$ or text messag$ or texting or computer$ or app or apps or (application$ adj3 phone$)) adj3 (psychotherap$ or psychiatr$ or counsel$)).ti,ab.

42. ((smartphone$ or smart-phone$ or smart phone$ or cellphone$ or cell-phone$ or cell phone$ or mobile phone$ or android$ or iPhone$ or sms messag$ or text messag$ or texting or computer$ or app or apps or (application$ adj3 phone$)) adj3 (depress$ or anxiety or anxious)).ti,ab.

43. ((smartphone$ or smart-phone$ or smart phone$ or cellphone$ or cell-phone$ or cell phone$ or mobile phone$ or android$ or iPhone$ or sms messag$ or text messag$ or texting or computer$ or app or apps or (application$ adj3 phone$)) adj3 health promot$).ti,ab.

44. (wearable adj3 (device$ or technolog$) adj3 (stress or burnout or burn-out)).ti,ab.

45. (wearables adj3 (problem solv$ or problem-solv$)).ti,ab.

46. (wearable adj3 (device$ or technolog$) adj3 (problem solv$ or problem-solv$)).ti,ab.

47. (wearables$ adj3 (problem solv$ or problem-solv$)).ti,ab.

48. (wearable adj3 (device$ or technolog$) adj3 (self help or self-help or selfhelp or selfcare or self-care or self care)).ti,ab.

49. (wearables adj3 (self help or self-help or selfhelp)).ti,ab.

50. (wearable adj3 (device$ or technolog$) adj3 (CBT or cognitive therap$ or cognitive behav$ therap$)).ti,ab.

51. (wearables adj3 (CBT or cognitive therap$ or cognitive behav$ therap$)).ti,ab.

52. (wearable adj3 (device$ or technolog$) adj3 mindfulness).ti,ab.

53. (wearables adj3 mindfulness).ti,ab.

54. (wearable adj3 (device$ or technolog$) adj3 (psychotherap$ or psychiatr$ or counsel$)).ti,ab.

55. (wearable adj3 (device$ or technolog$) adj3 (depress$ or anxiety or anxious)).ti,ab.

56. (wearables adj3 (psychotherap$ or psychiatr$ or counsel$)).ti,ab.

57. (wearables adj3 health promot$).ti,ab.

58. ((digital or decision aid$ or ehealth or e-health or ihealth or i-health or mhealth or m-health or online or on-line or internet-based or internet$ or web-based or web$ or email) adj3 (well-being or wellbeing or resilience or stress$ or mental health or depress$ or anxiety or anxious)).ti,ab.

59. ((smartphone$ or smart-phone$ or smart phone$ or cellphone$ or cell-phone$ or cell phone$ or mobile phone$ or android$ or iPhone$ or sms messag$ or text messag$ or texting or computer$ or app or apps or (application$ adj3 phone$)) adj3 (well-being or wellbeing or resilience or stress$ or mental health or depress$ or anxiety or anxious)).ti,ab.

60. (wearable adj3 (device$ or technolog$) adj3 (well-being or wellbeing or resilience or stress$ or mental health or depress$ or anxiety or anxious)).ti,ab.

61. 27 or 28 or 29 or 30 or 31 or 32 or 33 or 34 or 35 or 36 or 37 or 38 or 39 or 40 or 41 or 42 or 43 or 44 or 45 or 46 or 47 or 48 or 49 or 50 or 51 or 52 or 53 or 54 or 55 or 56 or 57 or 58 or 59 or 60

62. 26 or 61

63. exp Employee Engagement/

64. exp Reemployment/

65. exp Work Load/ or exp Work-Life Balance/

66. exp Employment Status/

67. exp Employee Absenteeism/ or exp Employee Productivity/

68. exp Occupational Health/

69. (employee$ or worker$).ti,ab.

70. (employment or occupation or work or workplace$ or worksite$).ti,ab.

71. (burn-out or burnout or work engagement or work-engagement or absenteeism or presenteeism).ti,ab.

72. (return-to-work or return to work).ti,ab.

73. (sick$ adj1 (absence or absent or leave or listed)).ti,ab.

74. 63 or 64 or 65 or 66 or 67 or 68 or 69 or 70 or 71 or 72 or 73

75. 62 and 74

76. (e-mental health or EMH).ti,ab.

77. 74 and 76

78. ((digital or decision aid$ or ehealth or e-health or mhealth or m-health or online or on-line or internet-based or internet* or web-based or web$) adj3 (intervention$ or treatment$)).ti,ab.

79. ((smartphone$ or smart-phone$ or smart phone$ or cellphone$ or cell-phone$ or cell phone$ or mobile phone$ or android$ or iPhone$ or sms messag$ or text messag$ or texting or computer$ or app or apps or (application$ adj3 phone$)) adj3 (intervention$ or treatment$)).ti,ab.

80. (wearable adj3 (device$ or technolog$) adj3 (intervention$ or treatment$)).ti,ab.

81. (wearables adj3 (intervention or treatment$)).ti,ab.

82. (78 or 79 or 80 or 81) and 74 and 8

83. ((digital or decision aid$ or ehealth or e-health or mhealth or m-health or online or on-line or internet-based or internet* or web-based or web$) adj3 (train$ or program$)).ti,ab.

84. ((smartphone$ or smart-phone$ or smart phone$ or cellphone$ or cell-phone$ or cell phone$ or mobile phone$ or android$ or iPhone$ or sms messag$ or text messag$ or texting or computer$ or app or apps or (application$ adj3 phone$)) adj3 (train$ or program$)).ti,ab.

85. (wearable adj3 (device$ or technolog$) adj3 (train$ or program$)).ti,ab.

86. (wearables adj3 (train$ or program$)).ti,ab.

87. (83 or 84 or 85 or 86) and 74 and 8

88. 75 or 77 or 82 or 87

89. clinical trials.sh.

90. (randomi#ed or randomi#ation or randomi#ing).ti,ab,id.

91. (RCT or at random or (random* adj3 (administ* or allocat* or assign* or class* or cluster* or control* or determine* or divide* or division or distribut* or expose* or fashion or number* or place* or recruit* or split or subsitut* or treat*))).ti,ab,id.

92. (control* and (trial or study or group) and (placebo or waitlist* or wait* list* or ((treatment or care) adj2 usual))).ti,ab,id,hw.

93. ((single or double or triple or treble) adj2 (blind* or mask* or dummy)).ti,ab,id.

94. (stepped wedge or (cluster adj2 random*)).ti,ab,id.

95. trial.ti.

96. placebo.ti,ab,id,hw.

97. treatment outcome.md.

98. treatment effectiveness evaluation.sh.

99. mental health program evaluation.sh.

100. 89 or 90 or 91 or 92 or 93 or 94 or 95 or 96 or 97 or 98 or 99

101. exp animals/ not humans.sh.

102. 100 not 101

103. 88 and 102
