# Supplementary material for: Effectiveness of tailored digital health interventions for mental health at the workplace: A systematic review of randomised controlled trials
Source: PLOS Digit Health. 2022 Oct 21;1(10):e0000123. doi: 10.1371/journal.pdig.0000123 (PMC9931277; doi:10.1371/journal.pdig.0000123)
Supplement: S1 Appendix — (ZIP) [file pdig.0000123.s001.zip › EMPOWER Search Strategy Final/EMPOWER Revised Medline FINAL.docx]

**EMPOWER Revised Medline**

Medline 535 (FINAL need to add to Endnote)

1. Mental Disorders/

2. Anxiety Disorders/

3. exp Depressive Disorder/

4. Depression/

5. Stress, Psychological/

6. Stress, Physiological/

7. Occupational Stress/

8. Professional Burnout/

9. 1 or 2 or 3 or 4 or 5 or 6 or 7 or 8

10. exp Computers/

11. Smartphone/ or Cell Phone/

12. Text messaging/

13. Internet/

14. Wearable Electronic Devices/

15. Electronic Mail/

16. Therapy, Computer-Assisted/

17. 10 or 11 or 12 or 13 or 14 or 15 or 16

18. Cognitive Behavioral Therapy/

19. Self Care/

20. Problem Solving/

21. Mindfulness/

22. Counseling/

23. Psychotherapy/

24. Psychiatry/

25. Health Promotion/

26. 18 or 19 or 20 or 21 or 22 or 23 or 24 or 25

27. 9 and 17 and 26

28. ((digital or decision aid$ or ehealth or e-health or ihealth or i-health or mhealth or m-health or online or on-line or internet-based or internet$ or web-based or web$ or e-mail) adj3 (stress or burnout or burn-out)).ti,ab.

29. ((digital or decision aid$ or ehealth or e-health or ihealth or i-health or mhealth or m-health or online or on-line or internet-based or internet$ or web-based or web$ or e-mail) adj3 (problem solv$ or problem-solv$)).ti,ab.

30. ((digital or decision aid$ or ehealth or e-health or ihealth or i-health or mhealth or m-health or online or on-line or internet-based or internet$ or web-based or web$ or email) adj3 (self help or self-help or selfhelp or self care or self-care or self care)).ti,ab.

31. ((digital or decision aid$ or ehealth or e-health or ihealth or i-health or mhealth or m-health or online or on-line or internet-based or internet$ or web-based or web$ or e-mail) adj3 (CBT or cognitive therap$ or cognitive behav$ therap$)).ti,ab.

32. (iCBT or icognitive or i-cognitive).ti,ab.

33. ((digital or decision aid$ or ehealth or e-health or ihealth or i-health or mhealth or m-health or online or on-line or internet-based or internet$ or web-based or web$ or email) adj3 mindfulness$).ti,ab.

34. ((digital or decision aid$ or ehealth or e-health or ihealth or i-health or mhealth or m-health or online or on-line or internet-based or internet$ or web-based or web$ or e-mail) adj3 (psychotherap$ or psychiatr$ or counsel$)).ti,ab.

35. ((digital or decision aid$ or ehealth or e-health or ihealth or i-health or mhealth or m-health or online or on-line or internet-based or internet$ or web-based or web$ or e-mail) adj3 (depress$ or anxiety or anxious)).ti,ab.

36. ((digital or decision aid$ or ehealth or e-health or ihealth or i-health or mhealth or m-health or online or on-line or internet-based or internet$ or web-based or web$ or e-mail) adj3 health promot$).ti,ab.

37. ((smartphone$ or smart-phone$ or smart phone$ or cellphone$ or cell-phone$ or cell phone$ or mobile phone$ or android$ or iPhone$ or sms messag$ or text messag$ or texting or computer$ or app or apps or (application$ adj3 phone$)) adj3 (stress or burnout or burn-out)).ti,ab.

38. ((smartphone$ or smart-phone$ or smart phone$ or cellphone$ or cell-phone$ or cell phone$ or mobile phone$ or android$ or iPhone$ or sms messag$ or text messag$ or texting or computer$ or app or apps or (application$ adj3 phone$)) adj3 (problem solv$ or problem-solv$)).ti,ab.

39. ((smartphone$ or smart-phone$ or smart phone$ or cellphone$ or cell-phone$ or cell phone$ or mobile phone$ or android$ or iPhone$ or sms messag$ or text messag$ or texting or computer$ or app or apps or (application$ adj3 phone$)) adj3 (self help or self-help or selfhelp or selfcare or self-care or self care)).ti,ab.

40. ((smartphone$ or smart-phone$ or smart phone$ or cellphone$ or cell-phone$ or cell phone$ or mobile phone$ or android$ or iPhone$ or sms messag$ or text messag$ or texting or computer$ or app or apps or (application$ adj3 phone$)) adj3 (CBT or cognitive therap$ or cognitive behav$ therap$)).ti,ab.

41. ((smartphone$ or smart-phone$ or smart phone$ or cellphone$ or cell-phone$ or cell phone$ or mobile phone$ or android$ or iPhone$ or sms messag$ or text messag$ or texting or computer$ or app or apps or (application$ adj3 phone$)) adj3 mindfulness).ti,ab.

42. ((smartphone$ or smart-phone$ or smart phone$ or cellphone$ or cell-phone$ or cell phone$ or mobile phone$ or android$ or iPhone$ or sms messag$ or text messag$ or texting or computer$ or app or apps or (application$ adj3 phone$)) adj3 (psychotherap$ or psychiatr$ or counsel$)).ti,ab.

43. ((smartphone$ or smart-phone$ or smart phone$ or cellphone$ or cell-phone$ or cell phone$ or mobile phone$ or android$ or iPhone$ or sms messag$ or text messag$ or texting or computer$ or app or apps or (application$ adj3 phone$)) adj3 (depress$ or anxiety or anxious)).ti,ab.

44. ((smartphone$ or smart-phone$ or smart phone$ or cellphone$ or cell-phone$ or cell phone$ or mobile phone$ or android$ or iPhone$ or sms messag$ or text messag$ or texting or computer$ or app or apps or (application$ adj3 phone$)) adj3 health promot$).ti,ab.

45. (wearable adj3 (device$ or technolog$) adj3 (stress or burnout or burn-out)).ti,ab.

46. (wearables adj3 (problem solv$ or problem-solv$)).ti,ab.

47. (wearable adj3 (device$ or technolog$) adj3 (problem solv$ or problem-solv$)).ti,ab.

48. (wearables$ adj3 (problem solv$ or problem-solv$)).ti,ab.

49. (wearable adj3 (device$ or technolog$) adj3 (self help or self-help or selfhelp or selfcare or self-care or self care)).ti,ab.

50. (wearables adj3 (self help or self-help or selfhelp)).ti,ab.

51. (wearable adj3 (device$ or technolog$) adj3 (CBT or cognitive therap$ or cognitive behav$ therap$)).ti,ab.

52. (wearables adj3 (CBT or cognitive therap$ or cognitive behav$ therap$)).ti,ab.

53. (wearable adj3 (device$ or technolog$) adj3 mindfulness).ti,ab.

54. (wearables adj3 mindfulness).ti,ab.

55. (wearable adj3 (device$ or technolog$) adj3 (psychotherap$ or psychiatr$ or counsel$)).ti,ab.

56. (wearable adj3 (device$ or technolog$) adj3 (depress$ or anxiety or anxious)).ti,ab.

57. (wearables adj3 (psychotherap$ or psychiatr$ or counsel$)).ti,ab.

58. (wearables adj3 health promot$).ti,ab.

59. ((digital or decision aid$ or ehealth or e-health or ihealth or i-health or mhealth or m-health or online or on-line or internet-based or internet$ or web-based or web$ or email) adj3 (well-being or wellbeing or resilience or stress$ or mental health or depress$ or anxiety or anxious)).ti,ab.

60. ((smartphone$ or smart-phone$ or smart phone$ or cellphone$ or cell-phone$ or cell phone$ or mobile phone$ or android$ or iPhone$ or sms messag$ or text messag$ or texting or computer$ or app or apps or (application$ adj3 phone$)) adj3 (well-being or wellbeing or resilience or stress$ or mental health or depress$ or anxiety or anxious)).ti,ab.

61. (wearable adj3 (device$ or technolog$) adj3 (well-being or wellbeing or resilience or stress$ or mental health or depress$ or anxiety or anxious)).ti,ab.

62. 28 or 29 or 30 or 31 or 32 or 33 or 34 or 35 or 36 or 37 or 38 or 39 or 40 or 41 or 42 or 43 or 44 or 45 or 46 or 47 or 48 or 49 or 50 or 51 or 52 or 53 or 54 or 55 or 56 or 57 or 58 or 59 or 60 or 61

63. 27 or 62

64. work/ or return to work/ or work engagement/

65. Employment/

66. Absenteeism/ or Sick Leave/

67. Occupational Health/ or Occupational Health Services/

68. exp occupational groups/

69. (employee$ or worker$).ti,ab.

70. (employment or occupation or work or workplace$ or worksite$).ti,ab.

71. (burn-out or burnout or work engagement or work-engagement or absenteeism or presenteeism).ti,ab.

72. (return-to-work or return to work).ti,ab.

73. (sick$ adj1 (absence or absent or leave or listed)).ti,ab.

74. 64 or 65 or 66 or 67 or 68 or 69 or 70 or 71 or 72 or 73

75. 63 and 74

76. (e-mental health or EMH).ti,ab.

77. 74 and 76

78. ((digital or decision aid$ or ehealth or e-health or mhealth or m-health or online or on-line or internet-based or internet* or web-based or web$) adj3 (intervention$ or treatment$)).ti,ab.

79. ((smartphone$ or smart-phone$ or smart phone$ or cellphone$ or cell-phone$ or cell phone$ or mobile phone$ or android$ or iPhone$ or sms messag$ or text messag$ or texting or computer$ or app or apps or (application$ adj3 phone$)) adj3 (intervention$ or treatment$)).ti,ab.

80. (wearable adj3 (device$ or technolog$) adj3 (intervention$ or treatment$)).ti,ab.

81. (wearables adj3 (intervention or treatment$)).ti,ab.

82. (78 or 79 or 80 or 81) and 74 and 9

83. ((digital or decision aid$ or ehealth or e-health or mhealth or m-health or online or on-line or internet-based or internet* or web-based or web$) adj3 (train$ or program$)).ti,ab.

84. ((smartphone$ or smart-phone$ or smart phone$ or cellphone$ or cell-phone$ or cell phone$ or mobile phone$ or android$ or iPhone$ or sms messag$ or text messag$ or texting or computer$ or app or apps or (application$ adj3 phone$)) adj3 (train$ or program$)).ti,ab.

85. (wearable adj3 (device$ or technolog$) adj3 (train$ or program$)).ti,ab.

86. (wearables adj3 (train$ or program$)).ti,ab.

87. (83 or 84 or 85 or 86) and 74 and 9

88. 75 or 77 or 82 or 87

89. randomized controlled trial.pt.

90. controlled clinical trial.pt.

91. randomized.ab.

92. placebo.ab.

93. clinical trials as topic.sh.

94. randomly.ab.

95. (stepped wedge or (cluster adj2 random$)).ti,ab.

96. trial.ti.

97. 89 or 90 or 91 or 92 or 93 or 94 or 95 or 96

98. exp animals/ not humans.sh.

99. 97 not 98

100. 88 and 99
