# Supplementary material for: Effectiveness of tailored digital health interventions for mental health at the workplace: A systematic review of randomised controlled trials
Source: PLOS Digit Health. 2022 Oct 21;1(10):e0000123. doi: 10.1371/journal.pdig.0000123 (PMC9931277; doi:10.1371/journal.pdig.0000123)
Supplement: S2 Appendix — (DOCX) [file pdig.0000123.s002.docx]

Appendix S2: Tailored interventions Table

| **Authors** | **Name of digital intervention** | **Type of tailoring** | **What measure do they use to tailor?** | **What is measured/reported?** | **General content** |
| --- | --- | --- | --- | --- | --- |
| Billings et al., 2008[35], USA | Stress and Mood Management | Tailored to individual, participants are encouraged to go through the program at their own pace and to explore the sections most relevant to their current circumstances | Embedded assessment instrument within the stress management module screening for substance misuse, anxious mood, and dysphoria. | Knowledge, Attitude Toward Psychological Help, Mood Management Self-Efficacy, Stress, Mood, Depression, Anxiety, Negative Coping, Binge Drinking Stage of Change, Work Productivity | Cognitive-behavioural techniques such as goal setting, problem solving, identifying and testing negative thoughts, relaxation |
| Bolier et al., 2014 & Ketelaar et al., 2013 [40, 41],Netherland | Workers' Health Surveillance (WHS) | Tailored choice of online interventions and personalized advice. The personalised feedback was followed by an invitation for a tailored offer of self-help EMH interventions, on the basis of an algorithm based on the specific symptoms and the work-relatedness of the symptom | Online screening and cut-off point for case identification | Impaired work functioning, distress, work-related fatigue, risky drinking behaviour, depression, anxiety and posttraumatic stress | Psyfit; a course aimed at enhancing well-being and mental fitness; Colour Your Life, aimed at tackling (subclinical) depressive symptoms ;Strong at work, aimed at reducing work stress and how to cope with it; Don't Panic Online, aimed at reducing panic symptoms; Drinking Less, aimed at the reduction of risky drinking |
| Bostock et al., 2016[39], UK | Sleepio App | Fully automated and highly interactive, with no human contact. Tailored by the programme’s algorithms to each individual’s characteristics, personal goals, sleep diary data and progress | Each individual’s characteristics, personal goals, sleep diary data and progress | Sleep Condition Indicator (SCI) scores, presenteeism, abseenteeism, anxiety, depression | Content based on validated CBT manuals, is presented by an animated virtual therapist (‘The Prof’) |
| Ebert et al., 2016[36], Germany | iSMI (GET.ON Stress) | Tailoring based on the participants characteristics or input | Participants characteristics or input | Stress, Depression, Anxiety, Insomnia, Worry, HRQOL, Emotion, Work engagement, absenteeism, presentism | Seven sessions composed of modules for psycho-education (session 1), problem-solving (sessions 2–3), emotion regulation (sessions 4–6), planning for the future (session 7)—and an optional booster session 4 weeks after completion of the iSMI (session 8). Additionally, participants were offered eight optional modules that were integrated into sessions 2–6, and could be chosen based on individual need and/or preference |
| Grime, 2004[37] , UK | Beating The Blues | Highly structured, prescriptive and problem orientated | Not reported | Anxiety, Depression, | 8 sessions, cognitive components explore automatic thoughts, thinking errors and distraction, challenging unhelpful thinking, core beliefs and attributional style |
| Volker et al., 2015[42], Netherland | Return@Work | Tailor-made to the individual employee, depending on the symptoms and cognitions about RTW of the employee | Symptoms and cognitions about RTW of the employee. the employess work through modules and were free to discuss with occupational physician (OP). Occupational physicians and employees met each other face to face on a regular basis. The occupational physicians received automated email messages that were based on a decision aid | First RTW, Full RTW, Remission, Response | 5 modules (psychoeducation, t cognitions with regard to RTW, problem-solving skills, pain and fatigue, relapse prevention) |
| Weber et al., 2019[38], Germany | Kelaa mental Resilience App | Personalized feedback on questionnaire scores (e.g. "what are my scores? What does this mean for me? What should I do about this?), as well as detailed feedback on sleep data | Questionnaires scores | Users can track their stress, wellbeing, and resilience via short in-app questionnaires using validated scientific measures" | Psychoed: (motivation, social skills, coping skills building/using, healthy life skills, time management, resource management, budgeting, sleep, scheduling) Active CBT/DBT: (gratitude lists, reality testing, mindfulness, thought stopping, communication skills, scheduling) |

RTW=Return to work, HRQOL= Health-related quality of life
